# Chondroitin sulfates and their binding molecules in the central nervous system

**DOI:** 10.1007/s10719-017-9761-z

**Published:** 2017-01-18

**Authors:** L Djerbal, H Lortat-Jacob, JCF Kwok

**Affiliations:** 1 Institut de Biologie Structurale, University Grenoble Alpes, CNRS, CEA, F-38027 Grenoble, France; 2 School of Biomedical Sciences, Faculty of Biological Sciences, University of Leeds, Leeds, LS2 9JT UK

**Keywords:** Proteoglycans, Glycosaminoglycans, Chondroitin sulfate, Protein-glycosaminoglycan interactions, Central nervous system, Plasticity, Perineuronal nets

## Abstract

Chondroitin sulfate (CS) is the most abundant glycosaminoglycan (GAG) in the central nervous system (CNS) matrix. Its sulfation and epimerization patterns give rise to different forms of CS, which enables it to interact specifically and with a significant affinity with various signalling molecules in the matrix including growth factors, receptors and guidance molecules. These interactions control numerous biological and pathological processes, during development and in adulthood. In this review, we describe the specific interactions of different families of proteins involved in various physiological and cognitive mechanisms with CSs in CNS matrix. A better understanding of these interactions could promote a development of inhibitors to treat neurodegenerative diseases.

## Introduction

**Fig. 1:**
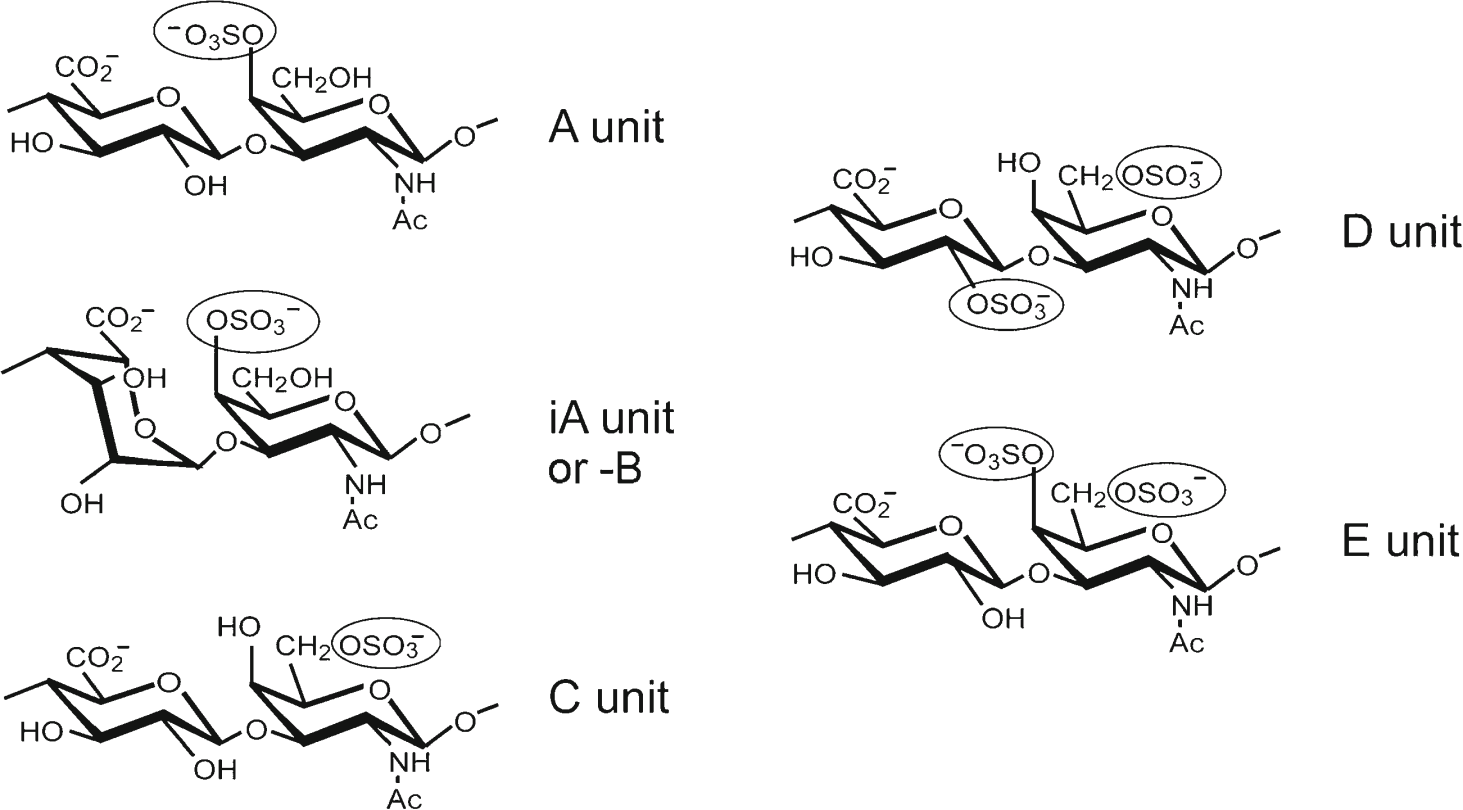
Structure of disaccharide units of chondroitin sulfate. Chondroitin sulfate consists of repeating disaccharide unit composed of D-glucuronic acid (GlcA) and *N*-acetylgalactosamine (GalNAc). Each monosaccharide may be sulfated on different residues. CS-A: carbon (C) 4 of the GalNAc. CS-C: C6 of the GalNAc. CS-D: C2 of the GlcA and C6 of the GalNAc. CS-E: C4 and C6 of Gal-NAc. GlcA can be epimerized into L-iduronic acid (iA unit) resulting in CS-B

Chondroitin sulfate (CS) is an important sulfated carbohydrate belonging to glycosaminoglycan family (GAG). CS was first obtained from cartilage by Fisher and Boedecker in 1861 and was isolated in purer form by Krukenburg in 1884. Seven years later, Schmiedeberg showed that it contains a hexosamine and hexuronic acid but the presence of a sulfuronic group was not mentioned at that time [1]. It was until 1915, Levene and Forge finally resolved the complete structure of CS [2–5]. CS is composed of a D-glucuronic acid (GlcA) and *N*-acetylgalactosamine (GalNAc) [1, 6]. Sulfation is one of the main modification on CSs. The sulfation is often added on C-4 and/or C-6 of GalNAc and/or C-2 of GlcA [7]. The sulfation position results in different forms of CS: CS-A, CS-C, CS-D, CS-E (Fig. 1). This sulfation pattern confers different roles to CSs and allows selective interactions with different molecules. Apart from sulfation, GlcA can be epimerized into L-iduronic acid (idoA) resulting in CS-B, which is also called dermatan sulfate (DS; Fig. 1).

Like most GAGs, CS is located in the extracellular matrix (ECM), at the cell surface or associated to the plasma membrane, in most animal tissues [8]. As such they appeared to be strategically positioned to control various important processes occurring at the cell-tissue interface. It is also found in the intracellular granules of certain cells like mast cells [8–10]. Both in the ECM and at the cell surface, CS is linked to a core protein to form chondroitin sulfate proteoglycan (CSPG). Localization of CSPGs in ECM make them more accessible for different molecules involving in different mechanisms [11].

The expression of CS is tissue dependent and it is present at a high level in the ECM in cartilage and central nervous system (CNS). CS constitutes the most abundant GAG in the cartilage [12]. Many studies have reported the positive effect of CS in treatment of osteoarthritis [13]. Indeed, CS inhibits the apoptosis of chondrocytes, metalloproteases degradation of cartilage, and activation of pro-inflammatory enzymes [14, 15]. CSPGs are also the major components of ECM in the CNS. They are critical for the formation, development and maintenance of brain morphology and function [16]. CSPGs are highly upregulated in glial scar after CNS injury and they inhibit axonal regeneration [16]. Recently, CSPGs have also been shown to control memory retention in mouse model of Alzheimer’s disease. Enzymatic removal of CSPGs enhances memory retention via enhanced plasticity, which would be useful in improving condition such as neurodegeneration [17, 18].

CS composition is also cell-type dependent and changes at different development stages. In nervous system, CS in the ECM changes during ontogenesis. While CS-C is the most expressed CS during embryogenesis, CS-A is the most abundant in adulthood [19, 20]. Apart from biochemical evidence using high performance liquid chromatography, these developmental changes in CSs are also confirmed using immunological techniques. Monoclonal antibodies are developed to specifically recognise different isoforms of CS chain. This tool provides the possibility of mapping the CS distribution during ontogenesis [21]. Furthermore, the spatio-temporal expression of CSs in brain ECM has been investigated. It has been shown that brain CSs exhibit structural diversity and developmental regulation, which suggests that CSs are implicated in diverse functions [22, 23].

The aim of this review is to provide an overview of all reported CS-interacting proteins, with respect to brain function, how they are involved in the maintenance of the ECM structure and their potential functional role. We focus on the interaction of proteins with various CS sulfation pattern and how these promote their signalling to accomplish their function including growth, differentiation, guidance and plasticity within the CNS.

## Organization of the CSPG network in the CNS

CSPG is the major component of ECM in the CNS representing as much as ~20% of its total volume [24]. While the ratio of total CS vs heparan sulfate (HS) in the CNS is 9:1, this ratio decreases to 7:3 in the perineuronal net (PNN) matrix [25].

CSPG extraction from the brain ECM, using a sequential method based on different buffers, revealed three distinct compartments: a diffuse matrix, a cell surface-associated matrix and a condensed matrix, which contains different types and amounts of CSPGs [25–27]. Almost all common CSPGs are found in all three compartments, which bear different ECM properties, but in different concentrations and with different molecular characteristics. The use of physiological saline allows the extraction of ECM molecules from the diffuse ECM. Western blotting analysis showed that the diffuse matrix extract contains all forms of neurocan, phosphacan (400 kDa) and brevican (145 kDa) and aggrecan (>500 kDa). The cell surface-associated matrix, which is released with detergent- or high-salt buffer contains membrane associated CSPG – NG2 and also the other CSPGs. Finally the condensed matrix assembly such as PNNs is extracted with 6 M urea buffer and contains almost similar species of neurocan, brevican and aggrecan, but without NG2. This compartment also contains a large amount of versican [25, 27]. Other than core proteins, the sulfation pattern of CS chains is also different in these compartments. Disaccharide analysis has revealed that CS-A is the major CS GAG in adult rat brain ECM and it is more abundant in the diffuse matrix, whereas, the disulfated and/or IdoA containing CSs including CS-B, CS-D and CS-E are more abundant in the PNNs [25].

**Fig. 2:**
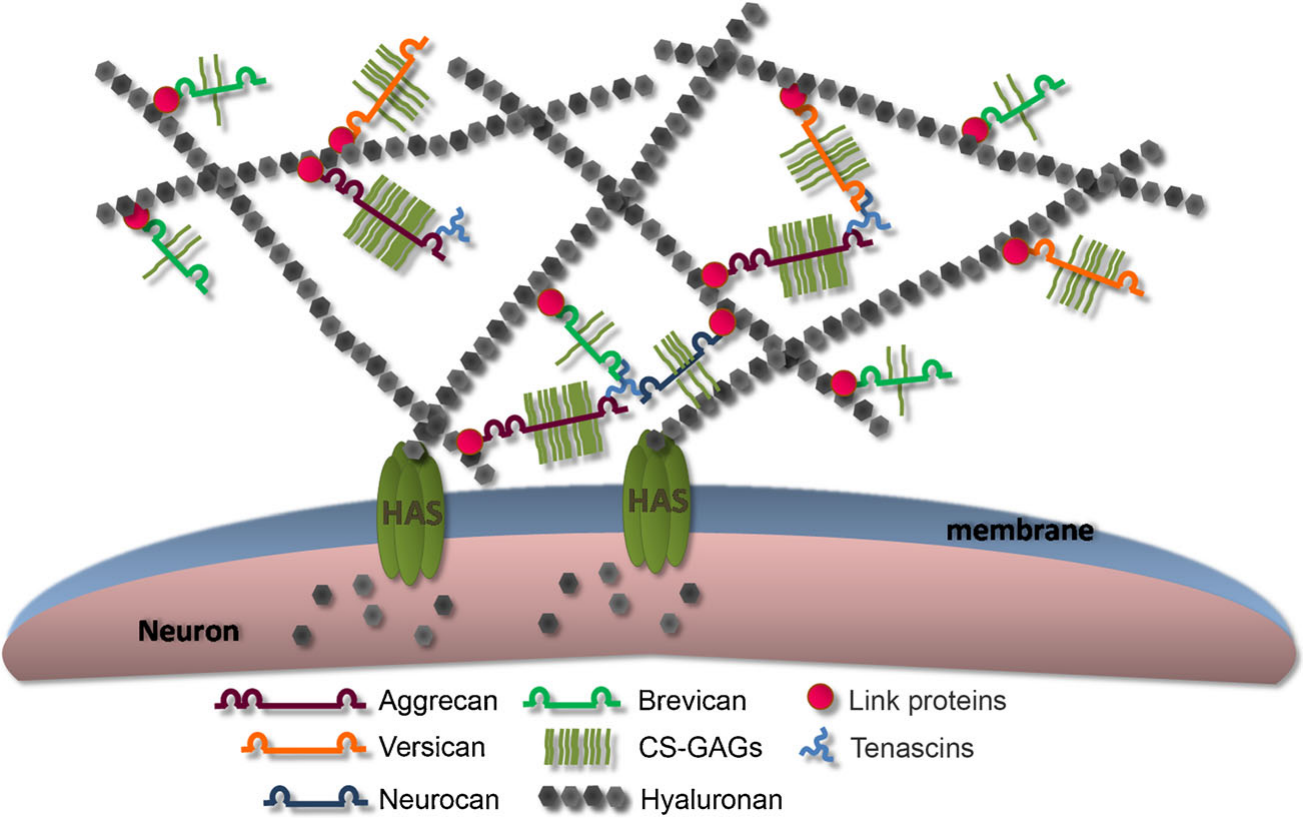
Structure of PNNs. PNN is composed mainly of CSPGs from the family of lectican (including aggrecan, versican, bervican and neurocan), hyaluronan (HA), link proteins and tenascin-R (Tn-R). HA is the backbone of PNN on which the CSPGs lay. It is anchored by a family of transmembrane enzymes, hyaluronan synthases (HASs). CSPGs bind to HA via a link protein. Tn-R is also involved in the organisation of PNNs by assembling of CSPGs at the C-termini (*Kwok et al. 2011)*

PNN is a highly organized ECM (Fig. 2) found mainly in parvalbumin (PV) positive GABAergic interneurons in the CNS although PNN is also observed in neurons without PV expression [28–31]. This macromolecular assembly, in addition to CSPGs, is composed of hyaluronan (HA), link proteins and tenascin-R (Tn-R). HA is the backbone of PNN on which CSPGs interact. It is synthetized by a transmembrane enzyme, hyaluronan synthase (HAS) and is responsible for anchoring PNN on the neuronal surface [32, 33]. Aggrecan is the key CSPG for PNN formation in the cortex although its role can be substituted by other CSPGs in other regions [33–35]. The interaction of CSPGs and HA is stabilised by a family of proteins called link protein, hyaluronan and proteoglycan-binding link proteins (HAPLNs) [36]. This molecular interaction is further stabilized by the trimeric Tn-R which can bind up to three CSPGs [37]. Both HAPLN and Tn-R are crucial for the stabilization and condensed nature of PNN [37, 38].

This unique molecular network looks optimally designed to accomplish very specific functions. It has been proposed that the immature PNNs act like a reservoir of molecules. It attracts the neurotrophic factors responsible for the survival and the growth of the neuronal cells [39]. Moreover, the appearance of the mature form of PNN coincides with the closure of the critical period, a period when experience-dependant plasticity is consolidated [40]. It has been proposed that PNN is important in stabilizing existing synapses and inhibiting further or aberrant synapses formation [38, 40]. In addition, PNNs are described as an ion exchanger in the brain. The two major components of the PNN, HA chain and CSPGs, are highly negatively charged, they can bind to the cations in the ECM and regulate the ion mobility [41].

## Proteins-GAGs interaction

GAGs interact with a large array of proteins to implement their functions [42, 43]. This interaction is of great importance to many physiological processes such as cell migration, growth, differentiation, guidance and development [44, 45]. They are also involved in pathological processes such as metastasis, neurodegeneration and inflammation [46–49]. Most GAGs are sulfated, including heparin (Hp), heparan sulfate (HS), keratan sulfate (KS), CS, DS and it was initially assumed that GAG-protein interaction is based on charge interaction. Multiple consensus amino acid sequences on the various GAG-binding proteins were later discovered on this basis [50]. Cardin and Weintraub identified that heparin binds to heparin-binding proteins through peptide sequences enriched in basic residues such as X-B-B-X-B-X and X-B-B-B-X-X-B-X where B is a basic residue and X is a hydropathic residue [51]. A third heparin specific sequence X-B-B-B-X-X-B-B-B-X-X-B-B-X was later reported, first in Willebrand factor and then in other proteins [52]. Further investigation has showed the importance of secondary and tertiary structure or the spatial distribution of basic residues. It has been shown, for example, that a distance of about 20 Ǻ frequently separates two basic amino acids in a number of heparin-binding peptides, facing opposite directions of an alpha-helix [53]. More recent work, however, also demonstrated that GAG binding sites can be well identified by considering neutral hydrogen bond donors, such as asparagine and glutamine, amino acids that importantly contribute to the specificity of the interaction [54]. For CSs, they bind to CNS proteins containing a specific motif rich in arginine and lysine, such as Otx2, which we shall discuss more in the later section [55, 56].

The specificity and selectivity do not confine to protein sequences, but are also dependent on the oligosaccharide sequence and the pattern of sulfation. Chemorepellent molecule semaphorin 3A is found to interact with CS-E and B, but not with CS-D even though these three CSs are all disulfated with the same charge over mass ratio per disaccharide unit [56, 57]. Selective binding of neurotrophic factors like midkine and BDNF to synthetic CS-E tetrasaccharide is observed and lead to neurite outgrouth. Whereas the binding of these factors to CS-R, a synthetic tetrasaccharide with the same number of sulfate groups as CS-E but distributed differently, is very weak and does not display a neuritogenic activity [58]. Also, different types of HS binds to different types of fibroblast growth factors (FGFs). For example, 2-O sulfate is required for the binding of FGF-2, 6-O sulfate is required for the binding of FGF-10 [59–62]. Recent evidence also shows that sulfation in CS/DS affects the binding and activation of FGF-2 [63–65]. These findings suggest the importance of sulfation pattern of GAGs in the specific binding to various proteins. Since HS is the most abundant GAG in the extracellular environment of many tissues, binding to HS/Hp is more documented than those to CS and KS. Most of interactions proteins-CSs has been characterized in the CNS.

## Interactions of CSs with different families of molecules in the CNS ECM

During development, immature neurons elongate their axons through a complex tissue structure to reach their appropriate synaptic partners located millimetres or even centimetres away. Diverse cellular and molecular mechanisms are adopted by embryos to guide the axons to their targets [66]. ECM molecules play a crucial role during this process through the involvement of either the ECM molecules or ECM-binding molecules [67]. CSs interact and cooperate with extracellular signalling proteins and receptors to modulate the axonal outgrowth. Removal of CSs by injection of chondroitinase ABC (ChABC) results in abnormal axonal outgrowth in zebrafish and rat embryos [68, 69] and disruption of retinal axons in mouse embryos [70]. Otherwise, CNS development and CNS injury share certain neural mechanisms including neural outgrowth, guidance and plasticity. Indeed, after CNS injury, the same molecules involved in development are up-regulated again, including CSPGs [71]. They form a chemical barrier, which inhibits axonal projection and regeneration [72]. Enzymatic removal of CSs using ChABC promotes functional recovery after spinal cord injury (SCI) in adult rats [73, 74].

**Fig. 3:**
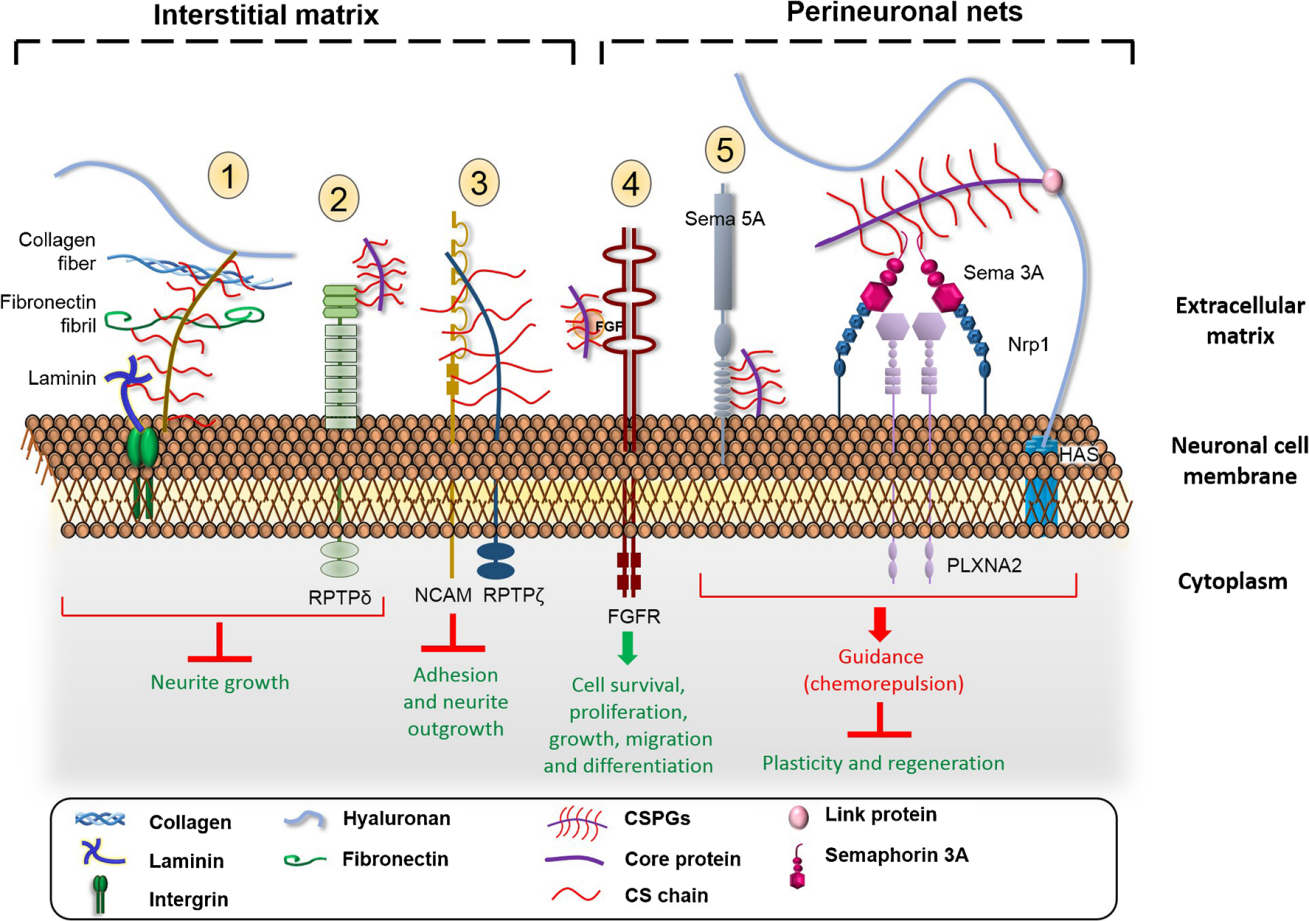
Interaction of CS glycan chains with different protein families in CNS matrix. CSPGs inhibit the growth cone via the interaction of its CS chain with 1) laminin and collagen and 2) bind its receptor protein tyrosine kinase (RPTP). 3) The neuronal adhesion molecule NCAM interacts with phosphacan (the extracellular part of RPTPζ) by its CS chain and results in an inhibition of adhesion and neurite growth. 4) CS, notably CS-E, acts as a binding partner of FGF to promote growth and differentiation. 5) An interaction of semaphorin 5 A (Sema 5A) with CS chain turns the attractive guidance protein into a repulsive cue. Semaphorin 3A (Sema 3A) is a repulsive guidance protein found in perineuronal nets and interacts specifically with CS-E motif. It exerts its chemorepulsive effect by signalling via plexin-neuropilin receptors. CS could be an additional constituent of sema 3A signalling complex

ECM in the CNS is a rich source of signalling molecules involved in different mechanisms of proliferation, differentiation, survival and migration of neurons. Activities and interactions of CSPGs with signalling molecules in ECM depend on many parameters: the core protein, the attached CS chains, their length, the degree and position of sulfates [75]. Composition of CSPGs varies in stages of development and physiological state, and this enables a large families of molecules, as described below, to interact with CSPGs via CSs chains to accomplish their functions (Fig. 3). The degree and position of sulfation on the CS chains confer different specific binding sites to various soluble factors in the ECM. Previous studies have reported an upregulation of CS-E unit and also chondroitin 6-sulfotransferase, an enzyme synthesising CS-C, after spinal cord injury [76, 77]. In the PNNs, CS-E are responsible for the binding of negative guidance molecule semaphorin 3A (Sema3A) and Otx2 [55, 57, 78, 79]. Recently, there are also studies describing the potential involvement of CS-C in epilepsy [80, 81]. These interactions lead to selective activation or inhibition of various signalling pathways. Here, we describe a families of proteins binding to CSs chain of CSPG to modulate the axonal outgrowth and guidance.

i) *Growth factors (GFs)*

GFs are biomolecules which support proliferation, growth, migration and differentiation [82–84]. In addition, they are involved in regulating metabolism, tissue repair and maintaining tissue homeostasis in adult organisms [85]. Some members exist ubiquitously in all tissues but some are expressed in a tissue-specific manner [86, 87]. GFs are usually secreted by neighbouring cells or at nerve terminals into the ECM and act locally due to their short life [88]. The secreted GFs bind to specific receptors on the surface of target cells and induce intracellular signalling pathway. Proteoglycans in the ECM bind to the GFs, facilitate the access and presentation of GFs to the receptor for subsequent signalling pathways [89]. During CNS development, many growth factors are shown to interact with CS chains, which we shall discuss below.

### Neurotrophic factors

are the family of GFs that promote survival, growth and differentiation of neuronal cells [90, 91]. Different neurotrophic factors, such as fibroblast growth factor, has been postulated to interact with heparin to promote the neurite outgrowth, hence their name “heparin-binding proteins” [92]. Interaction of neurotrophic factors with heparin/HS has attracted much attention similar to the involvement of HS in development [93]. However, these last decades more interest has been diverted to CSs notably in the CNS [94]. Here we describe heparin-binding proteins which interact with CSs in CNS.

### Fibroblast growth factors

(FGFs) are a large family of proteins, which exert a pleiotropic effects in different tissues. Basic fibroblast growth factor (bFGF) or FGF 2 is a multi-functional growth factor including its effect on survival of neurons and stimulate neurite extension [95]. It has been shown that bFGF binding to HS-, Hp-, or HA-bound surfaces stimulates neurite outgrowth from hippocampal neurons while the binding to CS- or DS-surfaces does not lead to the same observation [96]. On the other hand, Karumbaiah *et al.* has reported the potential of CS-A hydrogel enriched in bFGF in creating an endogenous niche for neural stem cells [97]. Surface plasmon resonance (SPR) analysis shows that CS-A from bovine trachea binds with high affinity to bFGF, brain-derived neurotrophic factor (BDNF) and interleukin 10 [97]. Screening of different GFs including FGF-2, −10, −16, −18 and Hp-binding epidermal growth factor-like growth factor (HB-EGF) against CS-E using filter binding assay, resonance mirror biosensor IAsys and GAG microarrays showed that these GFs bind to the CS-E from squid cartilage in a dose-dependent manner [64, 65]. FGF-16, FGF-18, and HB-EGF binds to CS-E (Kd ≈ 47.2, 8.9 and 16 nM, respectively) in a comparable affinity to the binding to Hp (Kd ≈ 34.7, 10.8 and 4.7 nM, respectively), while the affinity of FGF-2 and FGF-10 toward CS-E was lower than Hp [64, 65]. Screening of these GFs with oversulfated CS/DS hybrid chain purified from hagfish (CS-H) showed no binding by SPR, suggesting that the binding of the GFs to CS-E is a specific interaction but not due to non-specific charge interaction [98]. The binding of CS to FGF2 is important for the formation of neural spheres, proliferation and self-renewal of neural stem cells through the FGF2/MAPK pathway [99]. Removal of CSs using ChABC reduces neuronal proliferation and differentiation, and on the contrary, it increases the proliferation of astrocytes [100].

### Midkine and pleiotrophin

are two basic heparin-binding proteins localized in the radial glial fibres in embryonic brain, along which neural stem cells migrate [101]. They mediate neuronal cell adhesion and migration, and promote neurite outgrowth by interacting with cell surface heparin during development [102, 103]. It has been shown that CS-C inhibits the binding of pleiotrophin to its receptor 6B4 Proteoglycan/Phosphacan, which is an extracellular variant of receptor-like protein-tyrosine phosphatase (RPTP) ζ/RPTP β (this receptor family will be discussed in the next section), in rat brain. The binding of CS-C reduces pleiotrophin-induced neuronal migration along radial glial fibres. It has been postulated that CS and a portion of RPTPζ/RPTPβ could constitute the binding site of pleiotrophin [104, 105]. Similarly, Ueoka *et al.* have also shown that the adhesion of embryonic cortical neurons to midkine in culture is specifically inhibited by CS-E [106]. The interaction of midkine to CS-E is as strong as the binding to Hp [106]. The specific and direct interaction between CS-E/CS-H and midkine/pleiotrophin has also been shown by filter binding assay, IAsys and SPR [64, 98, 107]. Midkine and pleiotrophin are involved in neurodegenerative diseases such as Alzheimer’s disease. Deposition of midkine and pleiotrophin has been observed in the pathological senile plaque and/or neurofibrillary tangles in Alzheimer’s brain [102, 108]. LDL receptor-related protein (LRP) is a receptor of midkine and pleiotrophin and it is genetically linked to Alzheimer’s disease [102]. CS-Midkine/pleiotrophin interaction could be a potential target to treat these diseases. Thus, further understanding of this interaction should be performed. These findings suggest that CSs could be a binding partner or co-receptor for neurotrophic factors in the central nervous system.

ii) *Receptors*

### Receptor protein tyrosine phosphatases (RPTPs)

are a family of receptor-enzymes that remove phosphate from tyrosine in a protein. RPTPs are commonly found on growth cones [109] and are involved in the control of axon growth, guidance, regeneration and plasticity during development as well as after injury [110–112]. Indeed, a deficit of *O*-mannosylated RPTP ζ contributes to congenital muscular dystrophies [113–115].

Members of class II A RPTP including RPTPσ, RPTPδ and LAR (leukocyte common antigen-related phosphatase) bind with high affinity to CSPGs and HS proteoglycans (HSPGs) [116]. They are postulated to be the receptors of CSPG [117, 118]. RPTPσ knockout mice display a reduced CSPG inhibition after spinal cord injury and an enhanced regeneration after sciatic nerve crush injury [112, 119]. Mimetic peptide of RPTPσ wedge domain releases CSPG-mediated axonal inhibition *in vitro* by binding to RPTPσ and improve the functional recovery after SCI [120]. Binding of CSPG to RPTPσ induces a dephosphorylation of tropomyosin-related kinase B (TrkB), which leads to a down-regulation of dendritic spine formation [121]. TrkB is the BDNF receptor which, in contrast to CSPG, positively regulates the plasticity and spines formation in cortical neurons. Remarkably, binding of RPTPσ to CSPGs or HSPGs induces opposite effect on axonal growth. Cole *et al.* have shown that RPTPσ binding to HSPGs activates axonal growth while binding to CSPGs inhibits it [118]. The binding with CS chains inhibit the oligomerization of RPTPσ which are induced by HSPG [118].

LAR is another CSPG receptor [122]. It has been demonstrated by co-immunoprecipitation that LAR interacts with CSPG directly. This interaction leads to an inactivation of Akt and an activation of RhoA, thus inhibiting axonal growth [122]. Moreover, LAR knockout mice or mice treated with LAR-targeting peptides show an improvement of locomotor function after SCI [122, 123]. PG-RPTPs interaction may be a potential therapeutic target for functional recovery after CNS injuries.

### Nogo receptors NgR1 and NgR3

bind to Nogo and induce neurite outgrowth inhibition [124]. It has recently been reported that apart from Nogo, they can also bind to CSPGs and act as CSPGs receptors [125]. Both NgR1 and NgR3 bind specifically to disulfated CS-B, CS-D and CS-E with high affinity. The binding of CSPG to NgR1 and NgR3 inhibit neurite outgrowth. Double knockout of NgR1 and 3 shows increased regeneration after injury, and this is further enhanced with additional ablation of RPTPσ [125].

iii) *Adhesion molecules*

### Cell adhesion molecules (CAMs)

are surface glycoproteins mediating cell-cell and cell-extracellular interaction. The established connections between cells are important for maintaining tissue integrity and for cell communications [126]. Moreover, CAMs are fundamental for cell migration, notably during development of the CNS [127] and after traumatic brain injury [128]. Neural-CAM (N-CAM) is an adhesion molecule specific to the CNS. It has been implicated in various neuronal mechanisms. Indeed, this molecule is required for motor neuron sprouting, having thus a beneficial role in recovery after SCI. NCAM−/− mice show a significantly reduced locomotor recovery comparing the WT after SCI [129, 130]. N-CAM and neuron-glia CAM (Ng-CAM) bind with high affinity (Kd ≈ 0.5 nM) to the CSPG phosphacan [131]. Treatment with ChABC only reduces this binding by ~15% suggesting that the binding is mostly due to an interaction with the phosphacan core protein. The interaction of phosphacan and N-CAM or Ng-CAM leads to an reduced neurite outgrowth and adhesion *in vitro* [131]. Neurocan, another CSPG in the CNS matrix, also binds to N-CAM and Ng-CAM, and inhibits the neurite outgrowth [132]. Unlike phosphacan, ChABC treatment of neurocan significantly reduces this binding suggesting the interaction is mainly mediated through the CS GAG chains [132]. These findings suggest that N-CAM and Ng-CAM could be the receptors for the two CSPGs phosphacan and neurocan.

CAMs are also involved in other neuronal mechanisms in addition to neuronal migration. Contactin-1 is a glycosylphosphatidyl inositol (GPI)-linked membrane glycoprotein. It is a CAM and implicated in axonal growth, axonal and dendritic interactions of cerebellar interneurons and guidance [133]. With the use of SPR analysis, it has been shown that CS-E binds to contactin-1 with significant affinity (Kd ≈ 1.4 μM) and that this interaction is required for the neurogenesis mediated by CS-E [134].

iv) *Guidance proteins*

Guidance of dendrites and axons to their appropriate targets is a critical process for building a functional CNS during development [135]. These guidance molecules come from different families of proteins including secreted or cell surface guidance molecules and they can be attractive or repulsive [136].

### Semaphorin

is a large family of secreted and membrane-associated guidance molecules [137]. Initially being identified as repulsive guidance molecules, several studies in the last decades have reported a chemoattractive role of semaphorins [138–140]. Semaphorins guide the development of peripheral nerve projection and involved in synaptogenesis in the CNS [141, 142]. The persistence of their expression in adulthood suggests a role in the maintenance of pre-established connections and cerebral homeostasis [143].

Semaphorin 3A (Sema 3A) is one of the most studied members in class III semaphorins, which are upregulated after CNS injuries [144]. Owing to its ability in inducing growth cone collapse, Sema 3A is also called collapsin-1 and is the first member being identified in the semaphorin family [138]. It is a secreted protein, signaling via neuropilin-1(Nrp-1) and plexin (Plxn) receptors located at synapses [145–147]. During development, Sema 3A is expressed in a gradient across the cortical layers. It acts as a chemoattractive protein to guide the radial migration of cortical neurons [148]. In adult CNS, Sema 3A is found in the PNNs [78]. It modulates synapse morphology and function [147]. ChABC digestion reduces Sema 3A staining on the PNNs, suggesting an interaction between Sema 3A and CSPG [78]. Further investigations have shown that Sema 3A interacts with CS-E with high affinity [57]. Moreover, Nrp-1 can be modified post-translationally by CS chains, and this modification affects its ability to one of its effector VEGF [149]. These studies suggest that CSPGs could be additional constituents in the Sema 3A–Nrp-Plxn signalling complex. Moreover, Sema 3A is one of the most potent inhibitors to neuronal sprouting after SCI. It inhibits the axonal sprouting induced by nerve growth factor after SCI [150]. Indeed, Sema 3A could be one of the mechanisms which CSPG modulates plasticity. Targeting Sema 3A or its interaction with CSs could be a strategy to improve the plasticity after CNS trauma.

Semaphorin 5A (Sema 5A) is a membrane-associated semaphorin. Like other semaphorins, it is important in the development of the CNS [151]. Semaphorins class 5 is characterized by a specific domain containing two clusters of type-1 thrombospondin repeats (TSRs), which promote neurite outgrowth [152]. TSR displays a basic motif which can interact with the negative chain of HSPG and CSPG [153, 154]. Interestingly, the binding of Sema 5A to HSPG or CSPG triggers opposite responses. Sema 5A mediates neuronal attraction when it binds to HSPG and it becomes repulsive upon binding to CSPG [155]. This study indicates the proteoglycan-dependent function of Sema 5A.

Other than Sema 3A and 5A, Conrad *et al.* have tried to identify other ECM proteins which interact with CNS GAGs to promote the growth and differentiation of embryonic sensory nerve fibres using SPR and microarrays. The results indicate a significant interaction between CS-A and various guidance molecules, including Sema 3E, Sema 6B, ephrin A3 and Robo 2 [156].

Collapsin response mediator protein-4 (CRMP-4) is a 65 kDa phosphoprotein expressed in the CNS during development and in adulthood [157]. Dendrites extension of hippocampal neurons induced by Sema 3A is impaired in CRMP-4−/− hippocampal neurons, suggesting that CRMP-4 belongs to the Sema 3A signalling pathway which induces the growth cone collapse [158]. Moreover, CRMP-4 is identified as crucial protein that overcomes both axonal growth inhibition and scarring after SCI in adult mouse [158]. Interestingly, this intracellular protein interacts with CS. Indeed, CRMP-4 was purified using a CS affinity column [159, 160]. During early development in the CNS, active apoptosis which is essential for the normal development of CNS, causes the release of CRMP-4 into the extracellular space where it binds to CS [161, 162]. The downstream mechanism of the interaction between CRMP-4 and CS remains unknown but the above finding suggests an additional role of CNS ECM in sequestrating intracellular protein (s) released from apoptosis.

Orthodenticle homeobox protein 2 (Otx2) is another intracellular protein found interacting with CS in the ECM. Otx2 is a non-cell-autonomous transcription factor involved in brain morphogenesis [163]. It has been shown to interact with CSs in postnatal development and control plasticity [79]. Indeed, the role of Otx2 in plasticity has been investigated in the visual system in mice during the critical period. During this period, Otx2 is transferred from choroid plexus into the visual cortex and accumulates on the PNN of PV-cells [164]. Otx2 accelerates PV-cells maturation and PNNs formation. In return, PNNs concentrate Otx2 into the surface of PV-cells to be internalized [55]. A positive feedback loop between Otx2 and PNNs, during critical period as well as in maintaining PNN in adulthood, has been proposed [165]. Considering the important role of Otx2 and PNNs in controlling plasticity, further experiments have been performed to characterize the binding site of Otx2 in PNNs. Isothermal titration calorimetry (ITC) experiments show that a specific basic sequence in Otx2, rich in arginine and lysine, interacts specifically and with high affinity with CS-E and CS-D, low affinity with CS-C and not with CS-A [55]. This finding indicates, once more, that the interaction CS-protein is sulfation pattern-dependent, not charge dependent.

v) *Extracellular matrix proteins*

A number of fibrillar and glyco- proteins are also key components of the neuronal ECM in addition to CSPGs. They consist mainly of elastins, collagens, laminins and fibronectins [166]. Despite their small proportion in CNS matrix comparing to CSPGs, these proteins impose significant influence on the growth cone of neurons and regeneration [167, 168]. An addition of laminin to neurons in culture results in a drastic acceleration of neurite outgrowth [168]. Similarly, fibronectin promotes the neurite outgrowth and axonal regeneration of adult brain neurons *in vitro* [169]. Collagen VI protects the brain from neurodegeneration in ageing [170]. CSPGs and fibrous proteins have a complementary functions. Both work on the maintenance of the ECM homeostasis and the surrounding neurons. An implantation of a collagen/chondroitin 6-sulfate (CS-C) hydrogel containing embryonic striatal neurons allows the reconstruction of matrix and glial repair after a lesion in rat striatum [171]. It is likely that CSPGs and fibrous proteins interact with each other to accomplish their function. Astrochondrin, a CSPG on astrocyte surface and is involved in cerebellar granule cell migration, has been shown to interact specifically with laminin and collagen but not with fibronectin. Implication of CS chain of astrochondrin in this interaction is demonstrated by analysis of the astrochondrin binding to collagen in the presence of soluble CS in a radioligand binding assay. Indeed, soluble CSs are able to compete with astrochondrin to bind to collagen [172]. Snow *et al.* have reported that laminin and fibronectin are responsible for the inhibition effect of CSPGs on the growth cone [173].

Photomedin is another brain ECM protein, interacting with CS, less known comparing to fibronectin and laminin because it is less abundant and its spatiotemporal expression is restricted [174]. Photomedin 1 and 2 are identified as novel members of the olfactomedin family (OLF) [174]. OLFs are glycoproteins expressed mainly in the ECM of olfactory neuroepithelium, while photomedins are expressed mainly in retina ECM. They contain a specific sequence in their C-terminal, called OLF domain which has crucial implications in many neuronal mechanisms, including axonal growth and differentiation of chemosensory cilia [175]. ELISA experiments have shown that photomedins bind to CS-E with high affinity [174]. This is yet another evidence that CS-E interacts with different growth factors involved in neuronal migration and axonal guidance. Taken together, it may suggest that photomedins and growth factors could compete to bind to CS-E. Photomedins are proposed as a reservoir of CS-E and facilitates the localized action of growth factors [174].

vi) *Pathological protein*

Amyloid precursor protein (APP) is a transmembrane glycoprotein. APP undergoes a proteolytic processes giving rise to various peptides. Some of the resulting peptides are involved in neuronal plasticity and neurogenesis, but some of the others are pathological like amyloid beta peptide (Aβ) [176]. Indeed, Aβ is neurotoxic and found accumulated in neurons suffering from Alzheimer’s disease [177]. HSs and CSs have been reported to enhance the Aβ aggregation and the sulfate moieties on GAGs are the crucial key for this aggregation [178]. Recent study has reported that an overexpression of heparanase decreases the amyloid burden *in vivo* [179], and that PNN neurons resist neurotoxicity from Aβ and oxidative stress suggesting the neuroprotective role of PNNs [180, 181]. Otherwise, an interaction of APP and PG enhances the neurite outgrowth [182]. The direct interaction of CSPGs and APP has been demonstrated using solid phase binding assay. ChABC digestion of CS reduces the binding by 79%, this confirms the binding is through CS GAG chains [182].

While CSs/CSPGs are abundant in the CNS, study of PGs/GAGs interaction has been focused on HSPGs/HSs instead. Since amyloid beta peptide (Aβ) is accumulated in the extracellular space, which is rich in CSPGs, a specific and significant interaction could be suspected. Further investigation will shed light on the role of CSPG and CS chain in this disease. In addition, CS has been implicated in a number of other neuropathological conditions including epilepsy, stroke, schizophrenia, an in-depth understanding of the role of CS in these diseases will be crucial for targeting CS in the conditions [81, 183, 184].

## Conclusion

CS is the most abundant GAG in the CNS matrix. Its diverse pattern of sulfation and epimerization pattern allows precise controls of various physiological processes including the proper development of the CNS and the maintenance of neuronal homeostasis. Moreover, this diversity enables differential binding to a large family of proteins, with different affinities. This huge interaction between CSs and proteins places them at the first position in diverse signalling pathways. Thus, their involvement in various mechanisms during ontogenesis notably the growth cone, regeneration and plasticity.

Targeting the compositional change in CS as well as their interactions could be a promising approach to treat different pathologies. Chemical synthesis of CS oligosaccharides with defined sequences has recently made progresses [185, 186], opening the possibility to use those to manipulate a protein-CS interaction. Meanwhile, a better understanding of CS structure, their organization within the matrix, the mode of interaction with different types of proteins, are essential for targeting the important ECM component in the CNS.
